# Effects of Different Roughage Diets on Fattening Performance, Meat Quality, Fatty Acid Composition, and Rumen Microbe in Steers

**DOI:** 10.3389/fnut.2022.885069

**Published:** 2022-06-21

**Authors:** Xiaoyan Zhu, Boshuai Liu, Junnan Xiao, Ming Guo, Shumin Zhao, Menglin Hu, Yalei Cui, Defeng Li, Chengzhang Wang, Sen Ma, Yinghua Shi

**Affiliations:** ^1^Department of Animal Nutrition and Feed Science, College of Animal Science and Technology, Henan Agricultural University, Zhengzhou, China; ^2^Henan Key Laboratory of Grassland Resources Innovation and Utilization, Henan Agricultural University, Zhengzhou, China; ^3^Henan Herbage Engineering Technology Research Center, Henan Agricultural University, Zhengzhou, China

**Keywords:** wheat straw, alfalfa hay, peanut vine, meat quality, rumen microbe

## Abstract

This study aimed to evaluate different roughages on fatting performance, muscle fatty acids, rumen fermentation and rumen microbes of steers. Seventy-five Simmental crossbred steers were randomly divided into wheat straw group (WG), peanut vine group (PG) and alfalfa hay group (AG), with 5 replicates of 5 steers each. The results showed a highest average daily gain and lowest feed/gain ratio in AG group (*P* = 0.001). Steers fed alfalfa hay had the highest muscle marbling score and n-3 polyunsaturated fatty acid (PUFA), and also the rumen NH_3_-N and microbial protein (MCP) concentration among the three groups (*P* < 0.05). Correlation analysis showed that ruminal NH_3_-N and MCP were negatively correlated with muscle saturated fatty acid (SFA), while ruminal MCP was positively correlated with muscle PUFA and n-3 PUFA (*P* < 0.05). 16S rRNA analysis indicated that fed alfalfa hay decreased the abundance of *Ruminococcaceae_UCG-001*(*P* = 0.005). More importantly, muscle SFA deposition were positively correlated to the abundance of *Ruminococcaceae_UCG-001* (*P* < 0.05), while the muscle PUFA and n-3 PUFA deposition were negatively correlated to it (*P* < 0.01). Therefore, alfalfa hay provides a better fattening effect on steers. Alfalfa rich in n-3 PUFA would reduce the abundance of *Ruminococcaceae_UCG-001* involved in hydrogenation, increase the rumen protective effect of C18:3 *n*−3, which is beneficial to the deposition of muscle n-3 PUFA and PUFA.

## Introduction

Beef is considered to be an important source of protein and micronutrients, accounting for a high proportion in the diet of most Western countries, meanwhile the demand for beef is also increasing in China. Human diets in Western industrialized countries are usually characterized by high levels of saturated fatty acids (SFA) and *n*−6 polyunsaturated fatty acids (PUFA) and low levels of *n*−3 PUFA (1). The SFA contents in ruminant meats are as high as 50%, and fatty acids (FA) compositions in meat are closely related to human health (2). As consumers' pay more attention to food nutrition and health (3), they are worried that excessive consumption of SFA will lead to an increased risk of cardiovascular disease and cancer (4). Public health policies indicate that replacing SFA with PUFA, especially *n*−3 PUFA, should be beneficial to human health (5). People are very interested in improving the composition of FA, which mainly depends on rumen metabolism. Increasing the PUFA content of beef with current health recommendations, especially the *n*−3 PUFA, will further improve the nutritional characteristics and edible value of beef (5).

There are many factors affecting beef quality, but due to the regulatory effect of nutrition on fat deposition, the FA composition of beef may be altered by the nutritional background. Roughage is an indispensable component of ruminant diet, and usually accounting for 60–80% of the diet. In fact, the fermentation process of rumen microbes is greatly affected by roughage sources. Pitta et al. found that *Prevotella* (up to 33%) and *Rikenella-*like (up to 28%) genera were predominant genera in the rumen content of steers fed bermuda grass diet, while predominant genera were *Prevotella* (up to 56%) genus on the wheat diet (6). Using the 16S rRNA technology, Kong et al. found different species in the rumen bacterial communities of dairy cows fed alfalfa or triticale diets (7). Fernando et al. demonstrated two distinct rumen microbial populations in hay-fed and high-grain-fed steers, in which the hay-fed steers contained a significantly higher number of bacteria belonging to the phylum *Fibrobacteres*, whereas grain-fed cattle contained a significantly higher number of bacteria belonging to the phylum *Bacteroidetes* (8). The rumen fermentation parameters also changed with different dietary fiber source. For example, sheep fed with peanut vine had higher contents of propionate, butyrate, and total volatile fatty acids (VFA) in rumen than those fed with rape straw (9). Therefore, roughage is the key to effectively maintain rumen function (10), and rumen bacteria are involved in biological hydrogenation and isomerization *in vivo*, which may eventually affect the muscle FA deposition by changing the FA composition of the rumen digesta (11).

Wheat straw, peanut vine and alfalfa hay are different roughage sources, with different nutritional level and FA composition (Table 1). It was found that wheat straw has the lowest digestibility among the Gramineae straw, which is related to the lignin structure of wheat straw (12). After evaluation, the crude protein content of peanut vine was basically equivalent to that of alfalfa at full flowering stage (13), and can significantly improve the production performance and feed conversion rate of ruminants (14). Alfalfa with the characteristics of high protein and rich C18:3*n*−3, and has a remarkable effect on improving animal production performance (15), economic benefits and meat quality (16), especially the deposition of *n*−3 PUFA in muscles (17, 18). Forage interventions can impact the rumen environment and change the rumen microbial community (6, 11). Forages rich in C18:3*n*−3 have obvious effects on improving meat quality, promoting muscle C18:3*n*−3 and *n*−3 PUFA disposition (16). We hypothesized that forages with different nutritional levels and FA composition would selectively alter some rumen bacterial colonization, so as to improve the meat quality and PUFA composition. Considering the above, this study was designed to evaluate different roughages on fatting performance, meat quality, muscle FA, rumen fermentation and rumen microbes of steers. At the same time, in order to test the hypothesis, the correlations between rumen microbes and production performance, meat quality and FA, and rumen fermentation parameters were analyzed.

**Table 1:** Nutrient compositions of three roughages (DM %).

**Items^**a**^**	**Wheat straw**	**Peanut vine**	**Alfalfa hay**
DM	89.60	91.30	92.40
CP	5.60	12.20	16.80
EE	1.60	2.60	1.30
NDF	80.00	49.17	39.69
ADF	62.00	40.80	31.99
Ca	0.05	1.25	1.95
P	0.06	0.34	0.28
Myristic (C14:0)	4.34	2.67	1.05
Pentadecanoic (C15:0)	6.28	–	–
Palmitic (C16:0)	9.32	27.24	19.7
Palmitoleic (C16:1)	–	–	–
Heptadecanoic (C17:1)	0.54	–	0.43
Stearic (C18:0)	0.19	3.22	3.1
Oleic (C18:1*n*−9)	0.70	19.73	2.71
Linoleic (C18:2*n*−6)	1.05	31.14	18.4
Linolenic (C18:3*n*−3)	–	9.16	28.6
Arachidic (C20:0)	0.18	0.27	1.5

a *DM, dry matter; CP, crude protein; EE, ether extract; NDF, neutral detergent fiber; ADF, acid detergent fiber; Ca, calcium; P, phosphorus*.

## Materials and Methods

### Ethics Approval

All methods were carried out in accordance with relevant guidelines and regulations. The study was approved by the Animal Welfare and Ethics Committee of Henan Agricultural University (approval number: HENAU-2018-039). I confirmed that the study was reported according to ARRIVE guidelines, and did not have employed anesthesia or euthanasia methods inconsistent with the guidelines (e.g., chloral hydrate, ether, and chloroform). Permission was obtained from the farm owners before the samples were collected.

### Experimental Location, Animals, and Experimental Design

The equations should be inserted in editable format from the equation editor. The feeding experiment was carried out on the farm of Shuangmiao, which belongs to the company of HENGDU in Henan province. A total of 75 Simmental crossbred steers (body weight, 401.60 ± 14.76 kg) aged 12–14 months, were randomly divided into wheat straw group (WG: wheat straw + whole corn silage + concentrate), alfalfa hay group (AG: alfalfa hay + whole corn silage + concentrate), and peanut vine group (PG: peanut vine + whole corn silage + concentrate), with 5 replicates of 5 steers each, and each replicate was individually penned. The whole experiment lasted for 104 d, which consisted of a 7-d adaptation period, a 90-d feeding period and a 7-d sampling period. All steers were weighed and marked with numbered identification tags before the start of the experiment.

Steers in each group were fed a basal diet during the 7-d adaptation period and fed three kinds of trial diets during the 90-d feeding period. Diets in each period were prepared in the form of total mixed ration (TMR), and the cutting length and adding proportion of different roughages were consistent. The trial diets included 24.15% roughage, 27.55% whole corn silage and 48.3% concentrate, and satisfied the nutritional requirements of steers (19). Nutrient compositions of three roughages are shown in Table 1. Ingredients and chemical compositions of trial diets are shown in Table 2. Simmental crossbred steers were fed at 2.5% of body weight per day (8:00 and 16:00), with *ad libitum* access to water, and allowing 5–10% orts.

**Table 2:** Ingredients and chemical compositions of trial diets (DM%).

**Items**	**Treatments** ^a^		
	**WG**	**PG**	**AG**
**Ingredients**			
Wheat straw	24.15	0.00	0.00
Peanut vine	0.00	24.15	0.00
Alfalfa hay	0.00	0.00	24.15
Whole corn silage	27.55	27.55	27.55
Concentrate feed^b^	48.30	48.30	48.30
**Chemical compositions** ^c^			
NE_mf_, MJ/kg (DM)	6.48	6.73	6.76
CP	9.12	10.24	11.98
NDF	40.46	32.86	30.40
ADF	26.13	20.88	18.61
Ca	1.06	1.09	1.05
P	0.58	0.57	0.59

a *WG, wheat straw group; PG, peanut vine group; AG, alfalfa hay group; n = 5.*

a *The concentrate feed was consisted of 60% corn, 10% dry distiller's grains, 10% soybean meal, 13% wheat bran, 1% limestone, 2% NaHCO_3_ and 4% premix. The premix provided the following per kg of diets: Fe 1 200 mg, Zn 450 mg, Cu 150 mg, Se 5 mg, I 15 mg, Co 4 mg, VA 150 000 IU, VD_3_ 50 000 IU, VE 500 mg.*

c *NE_mf_was the calculated value, NE_mf_ = comprehensive net energy = maintain net energy (NE_m_) + net energy of weight gain (NE_g_); DM, dry matter; CP, crude protein; NDF, neutral detergent fiber; ADF, acid detergent fiber; Ca, calcium; P, phosphorus*.

### Sample Collection and Measurements

During d 1–d 98, offered feed and refusals in each steer were weighted and collected daily to calculate the average dry matter intake (DMI) of each steer. Individual body weight (BW) was measured before the morning feed on d 8 and d 98 to determine the body weight gain and average daily gain (ADG). The feed/gain ratio (F/G) was calculated by using the ratio of DMI to ADG in each steer. Rumen fluid samples were collected *via* a ruminal cannula 2 h after morning feed on the d 98 and the initial fluid was discarded before sample collection. Rumen fluid from each sample was mixed thoroughly and filtered through four-layer gauze, and pH values were measured immediately using a portable pH meter (testo206, Shanghai testo instruments international trade Co., Ltd). Triplicate of 5 mL rumen fluids from each sample were stabilized in 1 mL of 25% metaphosphoric acid solution and centrifuged at 3,000 × g for 15 min at 4°C, and cryopreserved at −20°C for further analysis of VFAs and ammoniacal nitrogen (NH_3_-N) concentrations. Three more 50 mL samples were frozen at −20°C for the determination of microbial protein (MCP) concentration. The last triplicate of 5-mL rumen fluids was frozen in a liquid nitrogen tank at −80°C for rumen bacterial 16S rRNA analysis.

Steers were slaughtered after the end of the 98-day feeding trial. Before being transported to HENGDU food factory, they were fasted for 24 h. According to the principle of animal welfare, steers were stunned electrically and bled to death by severing the jugular vein, and then immediately entered the assembly line for slaughtering and segmentation. After cooling at 0–2°C for 48 h, the carcasses were weighted to determine cold carcass weight (CCW). The dressing percentage (DP) was calculated by the following formulae: DP = (CCW/BW) × 100%, where the BW was the body weight of steer before slaughtering in factory. The meat and bones were separated from the body and weighed separately, which were used to calculate the net meat percentage (net meat mass/body weight × 100) and meat-bone ratio (meat mass/bone mass). The loin-eye area (cross-sectional area between the 6th and 7th rib on the left carcass) was used as a measure of carcass characteristic.

Longissimus dorsi muscle samples were collected from the left side of carcass (between the 6th and 7th rib) and divided into two parts. One part was used to measure pH value, marbling score, cooking yield and shear force, and other part was frozen at −20°C for further analysis of chemical composition and fatty acid composition.

### Chemical Analysis and Meat Quality

Experimental diets were dried in oven at 65°C for 48 h and crushed using a grinder with a 1 mm sieve before analysis. The contents of dry matter (DM), crude protein (CP), ether extract (EE), Ca and P were measured by methods No. 934.01, 990.03, 920.39, 985.35, and 986.24 of Association of Official Analytical Chemists (20). CP (*N* × 6.25) was analyzed according to the Kjeldahl method (Foss, Kjeltec 8400). The neutral detergent fiber (NDF) and acid detergent fiber (ADF) were examined using an Ankom Fiber Analyser (Ankom Technology, Fairport, NY) according to the method of Van Soest et al. (21). The pH value of longissimus dorsi muscle was measured at 48 h post-slaughter (pH 48) by a portable pH meter (testo205, Shanghai testo instruments international trade Co., Ltd), and the results were the average of the three points. Marbling score was determined visually by making use of a pink plate standard (Supplementary Figure 1). For cooking loss measurement, muscle samples were wrapped and weighed and placed in a thin plastic bag in water bath at 80°C. After 1 h, the cooked sample was taken out from the water bath, cooled at room temperature, blotted dry, and weighed again. Cooking loss was calculated as the percent weight lost relative to the initial sample weight. The meat samples used for the cooking loss determination were immediately cooled to 4°C and measured the share force in triplicate with a 1 cm-diameter Warner-Bratzler shear apparatus (C-LM 36, Harbin, China). The mean value was calculated for each muscle share force. The determination method of chemical components in muscle samples was the same as that of experimental diet.

The longissimus dorsi samples were directly methylated following the method of Blanco et al. (17). After the isolation of fatty acid methyl esters, samples were analyzed by gas chromatography equipped with a fame ionization detector (GC-FID; Shimadzu GC-2010 Plus, Japan) and a 100% cyanopropyl polysiloxane capillary column (SP 2560, 100 m, 0.25 mM i.d. and 0.20 μM film thickness, Sigma-Aldrich, St. Louis, MO). The injector and detector temperatures were maintained at 270 and 280°C, respectively. The initial column oven temperature was set at 100°C, held for 13 min, increased at 10°C/min to 180°C and kept for 6 min, increased at 1°C/min to 200°C and kept for 20 min, and then raised to 230°C at a rate of 4°C/min, remaining at that temperature for 10.5 min. Nitrogen was used as the carrier gas at a flow rate of 1.0 mL/min and 1.0 μL of sample was injected, and split ratio was 100:1. Peak areas were determined by comparing the retention times with the standards of fatty acid methyl esters (CDAB-CRM47885, Supelco, USA), and FA concentrations were calculated based on their peak areas. The composition of muscle SFA, monounsaturated fatty acid (MUFA), PUFA, total *n*−6, and *n*−3 PUFA, and the ratios of *n*−6 PUFA/*n*−3 PUFA were obtained from individual fatty acid percentages.

The rumen MCP concentration was measured using the spectrophotometric method according to the discription of Bradford (22). The concentration of rumen fluid NH_3_-N was determined using a colorimetric technique as reported by Bhandari et al. (23). The VFA concentration was quantified according to Zhang et al. (24) using a gas chromatography (HPGC, GC-2014, Shimadzu Corporation, Japan).

### DNA Extraction and 16S rRNA Sequencing

The total DNAs were extracted from the rumen fluid samples (*n* = 4) and performed according to the instructions of the E.Z.N.A^®^ DNA kit (OmegaBio-Tek, Norcross, GA, USA). The concentration of the extracted DNA was determined using a Nano Drop 2000 spectrophotometer (Thermo Scientific, Waltham, USA), and the quality of DNA extraction was detected by 2% agarose gel electrophoresis. The universal bacterial primers 338F (5'-ACTCCTACGGGAGGCAGCAG-3') and 806R (5'-GGACTACHVGGGTWTCTAAT-3') were used to amplify the V3–V4 region of the 16S rRNA gene from the extracted DNA. The amplification procedure was pre-denaturation at 95°C for 3 min, denaturation at 95°C for 30 s, annealing at 55°C for 20 s, extension at 72°C for 45 s, and after completing 27 cycles, 72°C extension for 10 min. The amplification reaction was 20 μL, 4 μL 5^*^FastPfu buffer, 2 μL 2.5 mM dNTP, 10 ng template amount, 0.8 μL forward primer, 0.8 μL reverse primer, 0.4 μL FastPfu polymerase. The PCR products were recovered using 2% agarose gel electrophoresis and purified using the DNA gel extraction kit (Axygen, China). The purified PCR product was quantified using Quanti Fluor^TM^-ST fluorimeter (Promega, China) to determine the DNA concentration. Three replicates of DNA extract from each sample were amplified and pooled in equimolar ratios into a single tube to construct PE libraries. Paired-end sequencing was conducted using the Illumina MiSeq platform (Meiji Biomedical Technology Co., Ltd, Shanghai, China) according to the manufacturer's instructions.

### Bioinformatics Analysis

Bioinformatics analysis was performed as described by Li et al. (25). According to the overlapping bases, the sequences at paired-end were spliced by using FLASH v1.2.7. Then, the low quality sequences were trimmed based on a minimum average mass fraction of 20. Barcode needs exactly matching. The overlap between paired-end reads with more than 10-bp and <2% mismatch was assembled into tags. The sequences were combined into operational taxonomic units (OTUs) using the clustering program UPARSE (version 7.1) based on 97% similarity. The Uchime algorithm carried out in Usearch software was used to eliminate chimeras. Representative sequences of each OTU were classified and annotated at various levels (phylum, genus) with Ribosomal Database Project (RDP) classifer algorithm and then aligned against entries in the Silva alignment database (SSU123) (26, 27). Alpha diversity including the Shannon, Sobs, and Chao richness indices were calculated using Mothur program (version v.1.34.0). The Kruskal-Wallis *H*-test method was used to identify differences between groups. A Principal Coordinate Analysis (PCoA) based on weighted UniFrac distances at the OTU level to generate two-dimensional plots, as to evaluate di?erences among samples.

### Statistical Analysis

All data were analyzed using one-way ANOVA (SPSS Statistic 24.0). Differences among means in different treatments were tested using Duncan's test, and the standard errors (SEM) from the analysis were shown. Correlation analyses were performed between performance, rumen fermentation, muscle FA deposition, and bacterial abundances by spearman correlation coefficient in R software. Statistical significance was declared at *P* < 0.05.

## Results

### Growth Performance

The results of growth performance are listed in Table 3. The DMI of steers fed peanut vine was significantly higher than those fed wheat straw and alfalfa hay (*P* = 0.001), but no significant difference was found between WG and AG group (*P* > 0.05). However, the ADG of steers fed alfalfa hay was significantly greater than those fed wheat straw and peanut vine (1.25 vs. 0.84 and 1.05 kg/d*, P* = 0.001). There was an effect of roughage diets on F/G, the lowest value of which was obtained by the steers consuming the diet with alfalfa (*P* = 0.001), whereas the F/G values did not differ among WG and PG groups (*P* > 0.05).

**Table 3:** Performance, carcass characteristics, chemical compositions and meat quality of longissimus dorsi muscle in Simmental crossbred steers (DM%).

**Items**	**Treatments** ^1^			**SEM**	***P*-value**
	**WG**	**PG**	**AG**		
**Performance** ^2^
DMI, kg/d	9.58^b^	10.17^a^	9.72^b^	0.08	0.001
ADG, kg/d	0.84^c^	1.05^b^	1.25^a^	0.05	0.001
F/G	11.41^a^	9.92^a^	7.80^b^	0.49	0.001
**Carcass characteristics**
Cold carcass weight, kg	256.10	261.10	276.90	4.63	0.161
Dressing percentage, %	53.35	52.69	54.12	0.78	0.781
Net meat mass, kg	215.17	218.69	233.73	4.11	0.147
Net meat percentage, %	44.83	44.13	45.68	0.69	0.686
Meat mass/Bone mass	5.25	5.16	5.42	0.06	0.283
Loin-eye area, cm^2^	76.50	74.75	79.40	1.79	0.598
**Chemical compositions** ^**3**^
Moisture, %	76.13	74.54	74.00	0.004	0.061
CP, %	19.72^b^	21.45^a^	20.40^ab^	0.30	0.040
EE, %	3.51	4.37	4.92	0.36	0.299
Ash, %	1.09	1.13	1.10	0.06	0.962
**Meat quality** ^4^
pH_48_	6.12	6.34	5.91	0.08	0.085
Marbling score	2.13^b^	2.20^b^	3.00^a^	0.16	0.037
Cooking yield, %	63.92	69.98	67.03	0.01	0.140
Shear force (*N*)	71.13	64.95	63.27	2.01	0.237

1 *WG, wheat straw group; PG, peanut vine group; AG, alfalfa hay group; n = 5.*

2 *DMI, dry matter intake; ADG, average daily gain; F/G, feed/gain ratio.*

3 *CP, crude protein; EE, ether extract.*

4 *PH_48_, the pH value of longissimus dorsi muscle at 48 h post-slaughter (pH 48).*

a,b,c *Means within rows with different superscript letters differ (P < 0.05).*

### Carcass Characteristics, Chemical Compositions, and Meat Quality

As shown in Table 3, three roughage diets had no significant effect on cold carcass weight, dressing percentage, net meat mass, net meat percentage, meat mass/bone mass, and loin-eye area (*P* > 0.05).

Moisture, EE and ash contents of meat were not influenced by three roughage diets (*P* > 0.05, Table 3), however, significant differences were detected in CP contents of meat (*P* = 0.04). The meat from steers fed wheat straw had the lowest CP content, which was 8.77 and 3.45% lower than that fed with peanut vine and alfalfa hay, respectively.

Different roughage diets had no effect on muscle pH48, cooking yield and shear force (*P* > 0.05, Table 3). However, steers fed alfalfa hay had the highest muscle marbling scores among the three groups (*P* = 0.037).

### Fatty Acids Composition of Longissimus Dorsi Muscle

The muscle fatty acid composition was different between WG, PG and AG group (Table 4). AG group had the least SFA in longissimus dorsi muscle (*P* = 0.035), which was 9.79 and 9.96% lower than that in WG group and PG group, respectively. The content of muscle n-3 PUFA in AG and PG group were significantly higher than that in WG group (*P* = 0.029). Simultaneously, AG group had the greatest muscle MUFA and PUFA, in which MUFA content was 4.50 and 7.16% higher than that in WG group and PG group, and PUFA content was 22.08 and 9.09% higher than that in WG group and PG group, respectively, but no difference was observed among the three groups (*P* = 0.109, *P* = 0.111).

**Table 4:** Fatty acid composition of longissimus dorsi muscle from Simmental crossbred steers fed different roughage diets.

**Items**	**Treatments** ^**1**^			**SEM**	***P*-value**
	**WG**	**PG**	**AG**		
Myristic (C14:0)	2.09	2.12	1.99	0.07	0.742
Palmitic (C16:0)	24.17	24.82	23.35	0.37	0.286
Palmitoleic (C16:1)	3.61	3.38	4.45	0.20	0.050
Heptadecenoic (C17:1)	0.74	0.77	0.83	0.02	0.246
Stearic (C18:0)	17.86	17.25	14.90	0.69	0.187
Oleic (C18:1*n*−9)	42.83	41.78	42.93	0.52	0.253
Linoleic (C18:2*n*−6)	6.25	6.96	7.83	0.30	0.091
Linolenic (C18:3*n*−3)	0.37^b^	0.45^a^	0.48^a^	0.02	0.029
Arachidic (C20:0)	0.28	0.28	0.21	0.02	0.393
Gadoleic (C20:1)	0.39	0.45	0.50	0.02	0.159
Arachidonic (C20:4*n*−6)	1.44	1.60	1.53	0.07	0.679
SFA^2^	44.41^a^	44.48^a^	40.45^b^	0.78	0.035
MUFA^3^	47.56	46.38	49.70	0.67	0.109
PUFA^4^	8.06	9.02	9.84	0.35	0.111
*n*−6 PUFA^5^	7.69	8.56	9.36	0.34	0.131
*n*−3 PUFA^6^	0.37^b^	0.45^a^	0.48^a^	0.02	0.029
*n*−6 PUFA/*n*−3 PUFA^5^^,^^6^	21.03	19.10	19.72	0.74	0.597

1 *WG, wheat straw group; PG, peanut vine group; AG, alfalfa hay group; n = 4.*

2 *SFA, saturated fatty acids (14:0 + 16:0 +18:0 + 20:0).*

3 *MUFA, monounsaturated fatty acids (16:1 + 17:1 + 9c18:1 + 20:1).*

4 *PUFA, polyunsaturated fatty acids (18:2n−6 + 18:3n−3 + 20:4n−6).*

5 *n−6 PUFA (18:2n−6 + 20:4n−6).*

6 *n−3 PUFA (18:3n−3).*

a,b *Means within rows with different superscript letters differ (P < 0.05).*

### Rumen Fermentation Characteristics

The parameters of rumen fermentation characteristics of Simmental crossbred steers are presented in Table 5. The pH values of rumen liquid were similar among the three treatments (*P* = 0.224), and all in the normal range. The concentrations of ruminal NH_3_-N and MCP in AG group were higher than those in WG and PG group (*P* = 0.001). Different dietary treatments had no effect on the concentrations of butyric acid (BA) and acetic acid (AA) (*P* = 0.068, *P* = 0.455), but the concentrations of propionic acid (PA) in PG and AG group were significantly higher than that in WG group (*P* = 0.002). Therefore, the AG group and PG group had the lower AA:PA ratio than of WG group (*P* < 0.001), which were beneficial to improve the rumen energy conversion efficiency.

**Table 5:** Effects of different roughage diets on rumen fermentation parameters in Simmental crossbred steers.

**Items^2^**	**Treatments** ^**1**^			**SEM**	***P*-value^4^**
	**WG**	**PG**	**AG**		
pH	6.74	6.45	6.63	0.07	0.224
NH_3_-N, mg/dl	4.59^c^	6.29^b^	8.01^a^	0.45	0.001
MCP, μg/ml	102.10^b^	118.63^b^	283.93^a^	30.60	0.001
**VFA** ^**3**^
AA, mg/ml	68.35	67.65	59.19	1.91	0.068
PA, mg/ml	10.35^b^	16.39^a^	16.09^a^	1.05	0.002
BA, mg/ml	9.93	10.87	11.18	0.43	0.455
AA/PA	6.61^a^	4.14^b^	3.69^b^	0.46	0.000

1 *WG, wheat straw group; PG, peanut vine group; AG, alfalfa hay group; n = 4.*

2 *MCP, microbial protein.*

3 *VFA, volatile fatty acids; AA, acetic acid; PA, propionic acid; BA, butyric acid; AA/PA, AA:PA ratio.*

4 *When P-values were below 0.001, the values were uniformly expressed as <0.001.*

a,b,c *Means within rows with different superscript letters differ (P < 0.05).*

### Rumen Microbial Diversities, Relative Abundance, and Bacteria Composition

**Figure 1:**
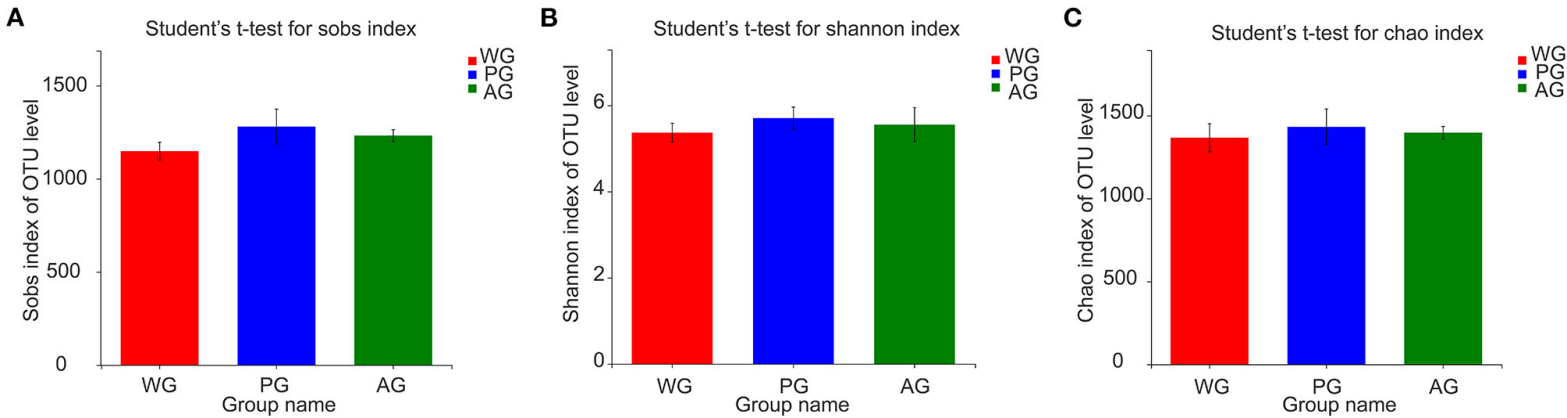
Alpha diversity statistics comparison. **(A)** Sobs index of OTU level. **(B)** Shannon index of OTU level. **(C)** Chao index of OTU level. Steers were fed a wheat straw diet (WG), *n* = 4; Steers were fed a peanut vine diet (PG), *n* = 4; Steers were fed an alfalfa hay diet (AG), *n* = 4. Error bars represent standard deviations and their lengths are adjusted at 95% confidence interval.

**Figure 2:**
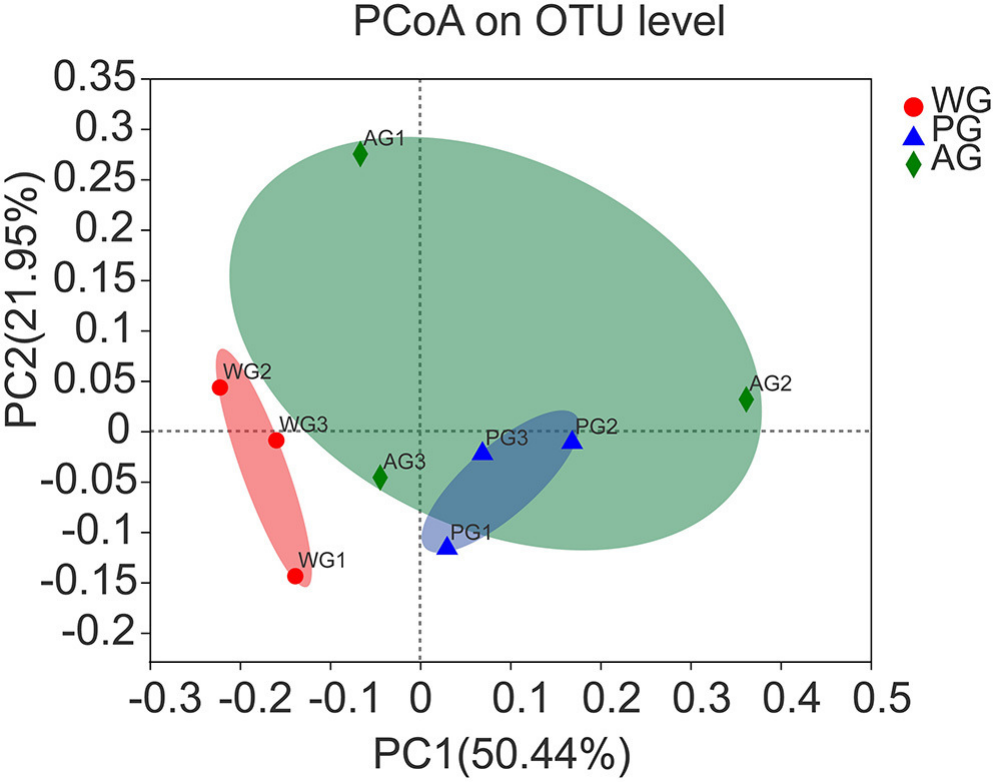
Principal coordinate analysis (PCoA) of rumen bacterial community structures of steers in the three groups. PCoA plots based on weighted UniFrac distance. Steers were fed a wheat straw diet (WG), *n* = 4; Steers were fed a peanut vine diet (PG), *n* = 4; Steers were fed alfalfa hay diet (AG), *n* = 4.

A total of 231,831 high quality sequences, with an average of 25,759 sequences per sample, were obtained after quality control and chimera removal. These qualified sequences were assigned to 1692 OTUs of rumen bacteria based on the 97% similarity level. Alpha diversity analysis results of the rumen bacteria under different treatments are presented in Figures 1A–C, which showed that Sobs, Shannon and Chao index had no significant changes, but an increasing trend of all indexes in AG and PG group (0.05 < *P* < 0.10). Meanwhile, the beta diversity was further shown by PCoA plots in Figure 2, with colors representing different roughage diets. PCoA plots constructed using the weighted UniFrac method and showed microbial clusters among the AG and PG group in principal components 1 and 2 (PC1:50.44%, PC2:21.95%), but the WG group distinct separation from AG and PG group, which indicated an effect of roughage diets in colonizing microbial communities.

**Figure 3:**
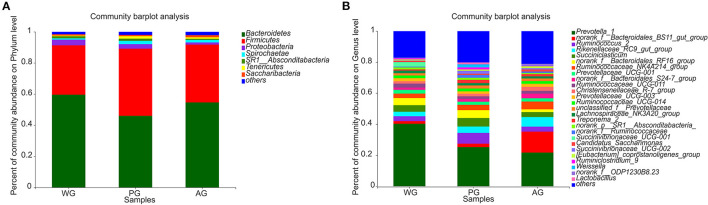
The relative abundance of phyla and genera in rumen bacteria community. **(A)** Community abundance on phylum level in three treatment groups. **(B)** Community abundance on genus level in three treatment groups. WG, wheat straw group, *n* = 4; PG, peanut vine group, *n* = 4; AG, alfalfa hay group, *n* = 4.

**Figure 4:**
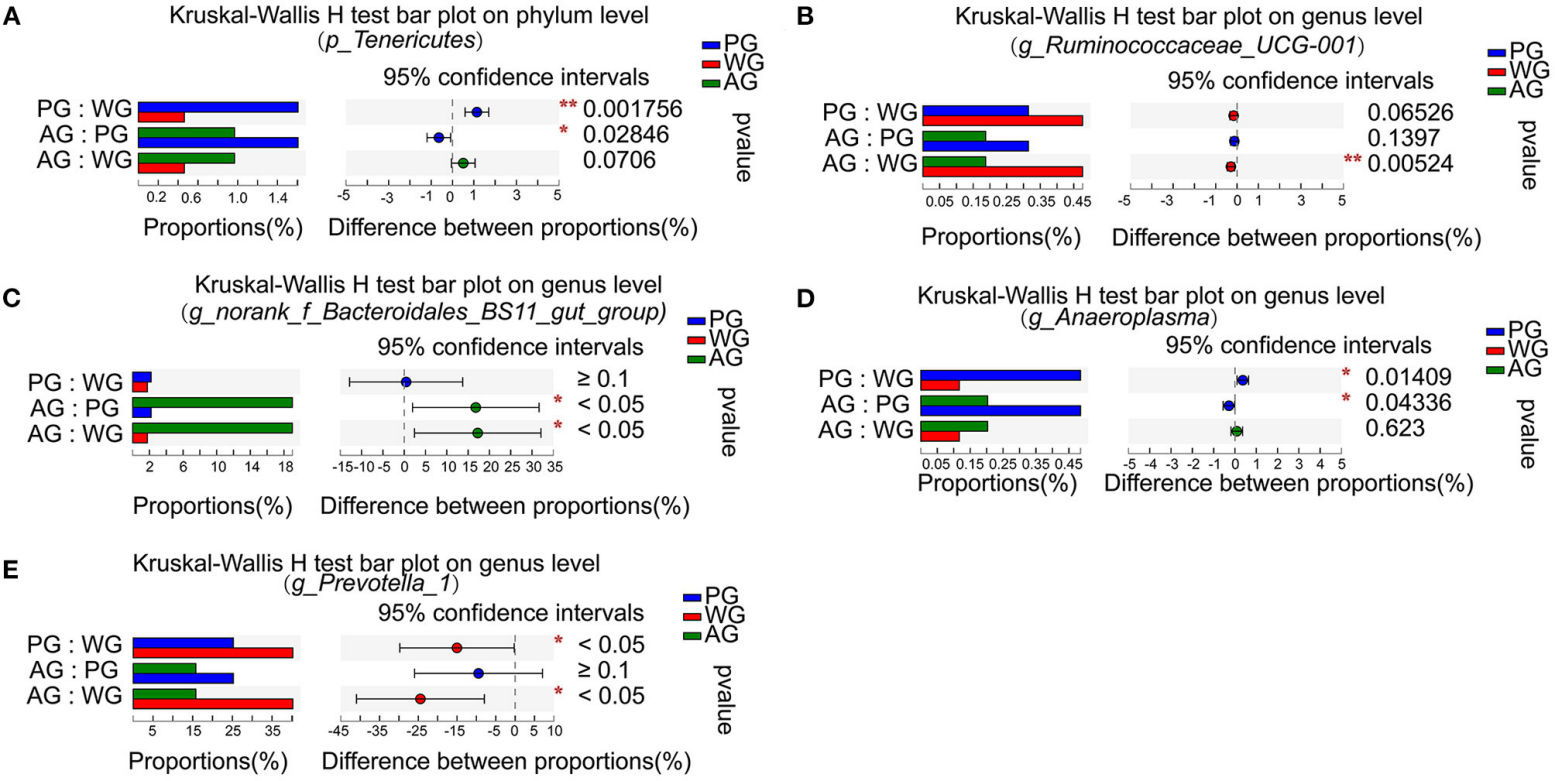
Difference of rumen bacteria at the phylum and genus level. **(A)** Analysis on the difference of *p_Tenericutes* abundance at phylum level. **(B)** Analysis on the difference of *g_Ruminococcaceae_UCG-001* abundance at genus level. **(C)** Analysis on the difference of *g_norank_f_Bacteroidales_BS11_gut_group* abundance at genus level. **(D)** Analysis on the difference of *g_ Anaeroplasma* abundance at genus level. **(E)** Analysis on the difference of *g_ Prevotella_1* abundance at genus level. WG, wheat straw group, *n* = 4; PG, peanut vine group, *n* = 4; AG, alfalfa hay group, *n* = 4. *P < 0.05, **P < 0.01.

Microbial compositions and differences at the phylum and genus levels are presented in Figures 3, 4. In all groups, the most dominant phyla were *Bacteroidetes* and *Firmicutes*, followed by *Proteobacteria*, with about 90% of sequences attributable to *Bacteroidetes* and *Firmicutes* (Figure 3A). The abundances of *Tenericutes* were affected by the dietary treatments, which in PG group was significantly higher than that in WG and AG group (*P* < 0.01, *P* < 0.05, Figure 4A). At the genus level, the most abundant genera were *Prevotella, Bacteroidales, Ruminococcus*, and *Succiniclasticum*, which accounted for 27.5–46.2, 7.8–18.2, 10.2–46.2, 2.9–6.2, and 3.2–7.4% of the bacterial community, respectively (Figure 3B).

Microbial differences analysis among three treatments showed that the abundances of ruminal *Ruminococcaceae_UCG-001* were lower in AG group than in WG group (*P* < 0.01, Figure 4B), but the abundances of *norank_f_Bacteroidales_BS11_gut_group* were higher in AG group than in WG and PG group (*P* < 0.05, Figure 4C). Compared with AG and WG diet treatments, the PG diet treatment had increased abundances of *Anaeroplasma* (*P* < 0.05, Figure 4D). The abundances of *Prevotella_1* were decreased with the AG and PG diet treatments compared to those under the WG condition (*P* < 0.05, Figure 4E). Therefore, the results indicated that ruminal bacteria changes in steers were affected by roughage sources.

### Correlation Analysis Between Muscle FA Deposition, Rumen Bacterial Genus Abundance, and Physiological/Production Parameters

The correlation analysis results between rumen fermentation parameters and muscle FA deposition are shown in Table 6. Ruminal NH_3_-N concentration was negatively correlated with SFA (*P* < 0.05), and significantly positively correlated with MUFA (*P* < 0.01). Ruminal MCP content was negatively correlated with SFA (*P* < 0.05), and positively correlated with muscle PUFA, *n*−6 PUFA, and *n*−3 PUFA (*P* < 0.05). The concentration of ruminal AA was positively correlated with muscle SFA (*P* < 0.05), and significantly negatively correlated with muscle MUFA (*P* < 0.01). In addition, the ratio of AA/PA was positively correlated with muscle SFA (*P* < 0.05), and negatively correlated with muscle MUFA and n-3 PUFA content (*P* < 0.05).

**Table 6:** Correlation between rumen fermentation parameters and muscle fatty acids.

**Items^a^**	**Rumen fermentation parameters** ^**b**^					
	**NH_**3**_-N**	**MCP**	**AA**	**PA**	**BA**	**AA/PA**
SFA	−0.737^*^	−0.721^*^	0.738^*^	−0.643	−0.472	0.789^*^
MUFA	0.840^**^	0.517	−0.910^**^	0.578	0.451	−0.780^*^
PUFA	0.350	0.718^*^	−0.317	0.447	0.326	−0.495
*n*−6 PUFA	0.329	0.698^*^	−0.299	0.431	0.345	−0.473
*n*−3 PUFA	0.586	0.830^*^	−0.515	0.576	−0.061	−0.709^*^
*n*−6/*n*−3	−0.480	−0.361	0.366	−0.438	0.402	0.558

a *SFA, saturated fatty acids (14:0 + 16:0 +18:0 + 20:0); MUFA, monounsaturated fatty acids (16:1 + 17:1 + 9c18:1 + 20:1); PUFA, polyunsaturated fatty acids (18:2n−6 + 18:3n−3 + 20:4n−6); n−6 PUFA (18:2n−6 + 20:4n−6); n−3 PUFA (18:3n−3).*

b *MCP, microbial protein; AA, acetic acid; PA, propionic acid; BA, butyric acid; AA/PA, AA:PA ratio.*

** *Indicate highly significant correlation (P < 0.01);*

* *indicate significant correlation (P < 0.05).*

**Figure 5:**
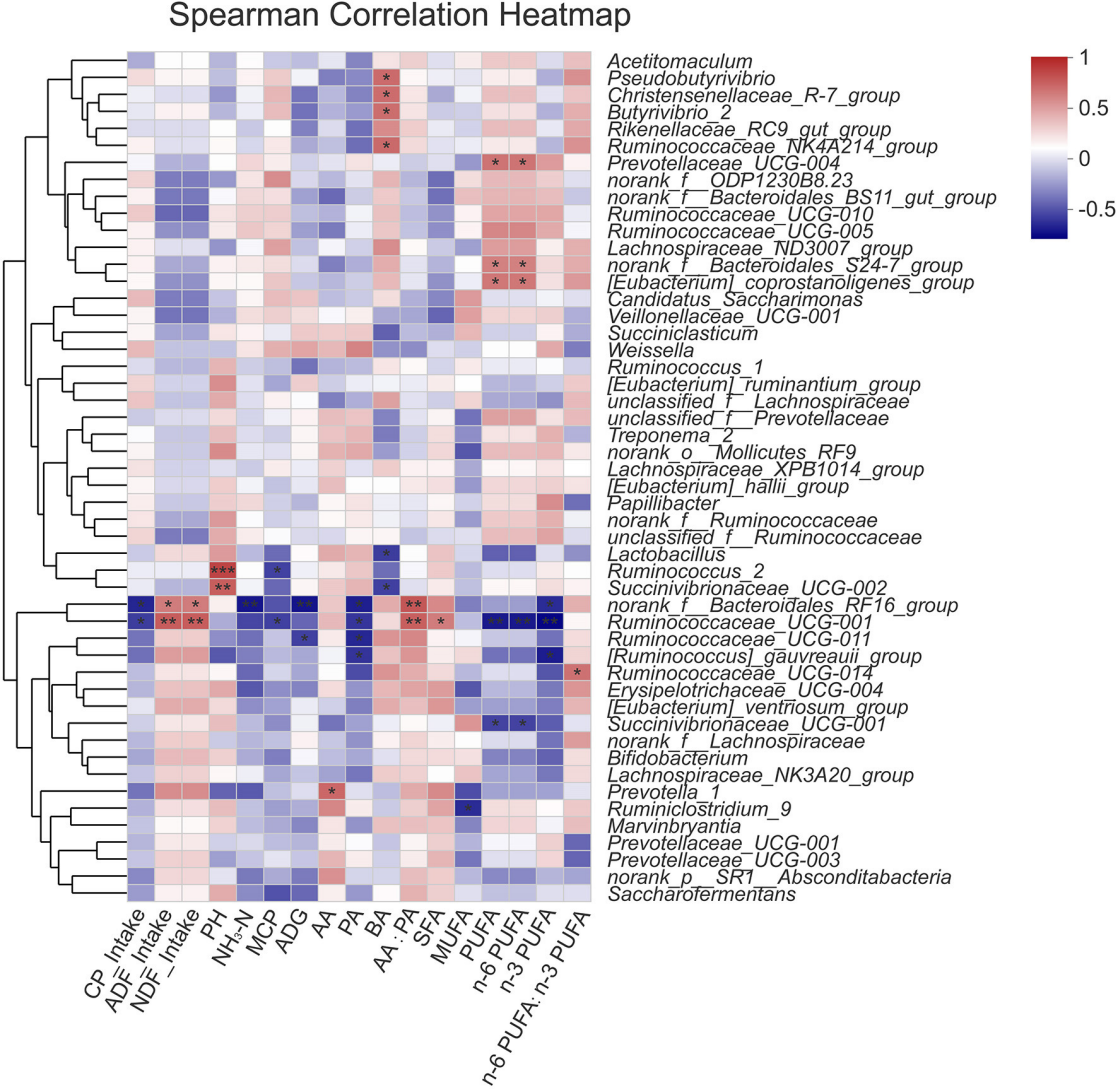
Correlation between rumen microorganism and environmental factors. Red stands for positive correlation, blue stands for negative correlation. SFA, saturated fatty acids (14:0 + 16:0 +18:0 + 20:0); MUFA, monounsaturated fatty acids (16:1 + 17:1 + 9c18:1 + 20:1); PUFA, polyunsaturated fatty acids (18:2*n*−6 + 18:3*n*−3 + 20:4*n*−6); *n*−6 PUFA (18:2*n*−6 + 20:4*n*−6); *n*−3 PUFA (18:3*n*−3). *P < 0.05, **P < 0.01, ***P < 0.001.

The relationships between 50 rumen bacterial genus abundance and physiological/production parameters (VFA, pH, NH_3_-N, MCP, FA and nutrient intakes) were evaluated in this study (Figure 5). The results showed that the relative abundance of *norank_f_Bacteroidales_RF16_group* and *Ruminococcaceae UCG-001* correlated negatively with CP intake (*P* < 0.05), and positively with ADF and NDF intake (*P* < 0.05, *P* < 0.01). The ruminal pH correlated positively with the abundance of *Ruminococcus_2* and *Succinivibrionaceae_UCG-002* (*P* < 0.01). The concentration of ruminal NH_3_-N correlated negatively with the abundance of *norank_f_Bacteroidales_RF16_group* (*P* < 0.01). The concentration of ruminal MCP correlated negatively with the abundance of *Ruminococcus_2* and *Ruminococcaceae UCG-001* (*P* < 0.05). The concentration of AA correlated positively with the abundance of *Prevotella_1* (*P* < 0.05). The concentration of PA correlated negatively with the abundance of *norank_f_Bacteroidales_RF16_group, Ruminococcaceae_UCG-001, Ruminococcaceae_UCG-011*, and *[Ruminococcus]_gauvreauii_group* (*P* < 0.05). The concentration of BA correlated positively with the abundance of *Pseudobutyrivibrio, Christensenellaceae_R-7_group, Butyrivibrio_2, Ruminococcaceae_NK4A214_group* (*P* < 0.05), and negatively with the abundance of *Lactobacillus, Succinivibrionaceae_UCG-002* (*P* < 0.05). The ratio of AA: PA correlated positively with the abundance of *norank_f_Bacteroidales_RF16_group* and *Ruminococcaceae UCG-001* (*P* < 0.01).

The abundance of *Ruminococcaceae UCG-001* exhibited a positive correlation with muscle SFA deposition (*P* < 0.05), and the abundance of *Ruminiclostridium_9* exhibited a negative correlation with muscle MUFA deposition (*P* < 0.05). *Prevotellaceae_UCG-004, norank_f_Bacteroidales_S24-7_group*, and *[Eubacterium]_coprostanoligenes_group* displayed positive correlations with muscle PUFA and n-6 PUFA deposition (*P* < 0.05), while *Ruminococcaceae UCG-001, Succinivibrionaceae_UCG-001* displayed negative correlations with muscle PUFA and *n*−6 PUFA deposition (*P* < 0.01, *P* < 0.05). The abundance of *norank_f_Bacteroidales_RF16_group, Ruminococcaceae UCG-001* and *[Ruminococcus]_gauvreauii_group* exhibited negative correlations with muscle *n*−3 PUFA deposition (*P* < 0.05, *P* < 0.01, *P* < 0.05). The ratio of n-6 PUFA: n-3PUFA correlated positively with the abundance of *Ruminococcaceae UCG-014* (*P* < 0.05). The above results demonstrated that rumen microbes were correlated with fatty acid contents of muscle in steers.

## Discussion

ADG and feed conversion are two important indexes to evaluate the production performance of steers, and the production performance mainly depends on the quality of feed provided. In this study, the higher ADG and better feed conversion were obtained by the steers fed with alfalfa hay as the roughage source diet, which were likely attributable to higher CP and lower NDF contents of alfalfa hay. Slaughter performance is an important index to evaluate the growth performance of meat animals (28). In the present study, the three roughage diets had no significant effects on slaughter performance, but the cold carcass weight of steers fed alfalfa hay increased, and the dressing percentage, net meat mass, meat mass/bone mass and Loin-eye area also increased. The above results still showed that alfalfa hay was an excellent roughage source and play a good role in improving the fattening effect and slaughter performance of Simmental crossbred steers.

Beef quality is essential in beef consumption. The changes of muscle nutrient content, especially fat and crude protein content, directly affect the quality and nutritional characteristics of meat (29). In the present experiment, the muscle protein in PG group was the best, followed by AG group, but no significant difference was observed between the two groups. In addition, the muscle fat deposition was highest (4.92%) in AG group and was 40.2 and 12.6% higher than that of WG and PG groups, respectively. The improvement of CP and fat deposition for AG group could be related to alfalfa nutritional composition and rumen fermentation. The pH is a key parameter to measure meat quality and has a direct effect on tenderness, cooking loss and shelf life of beef (30). Of course, the pH48 values of the three treatment groups were within the normal range, which ensured the reliability of production. Consequently, feeding alfalfa hay to Simmental crossbred steers during fattening period has the potential to produce high quality beef.

The composition of FA in meat is considered to be important for human health (31). It is believed that a reduction in the intake of SFA together with an increase in the consumption of PUFA may decrease the occurrence of cardiovascular diseases (CVD) in humans (5). The FA composition of meat can be changed easily by diet, especially with respect to the contents of C18:2*n*−6, C18:3*n*−3, and long-chain PUFA, because the dietary nutrients are deposited in tissue lipids in a more direct way as well as the result of rumen biohydrogenation of dietary lipids (32). The fatty acids in forages are mainly present as C18:3*n*−3 and this high proportion of C18:3*n*−3 can have an important influence on the fatty acid profiles of ruminant products (33). As expected, alfalfa hay diet increased the intramuscular contents of C18:2*n*−6 and C18:3*n*−3 in steers in the present study. In addition, the total SFA content in muscle decreased and total MUFA and PUFA contents increased when steers consumed the diets containing alfalfa hay, which were mainly caused by the changes of C16:1, C18:2*n*−6 and C18:3*n*−3 content. This result can be explained by the rich content of C18:2*n*−6 and C18:3*n*−3 in alfalfa, which is conducive to its deposition in muscle. Thus, alfalfa hay diet is considered to be an important factor of unsaturated fatty acid deposition in muscle, especially C18:2*n*−6 and C18:3*n*−3 deposition, which is also an important reason for the improvement of beef quality.

All the rumen pH values observed in this study were in the normal range and can be considered sufficiently high to maintain normal rumen fermentation. Rumen NH_3_-N is the degradation product of feed protein and is positively correlated with protein intake (34). In ruminal fluid, the greatest NH_3_-N concentration was found in AG group, followed by PG group, which resulted from the relatively higher CP content in alfalfa hay and peanut vine diet than that in wheat straw diet (35). Moreover, alfalfa contains more bioactive substances such as saponins that appear to affect specific rumen protozoa and bacteria, which may alter the rumen metabolism beneficially and increase the efficiency of microbial protein synthesis (36). High content of rumen NH_3_-N could stimulate rumen microbial growth and ultimately allowed MCP synthesis to increase (37), which could explain the highest MCP yield in steers fed alfalfa hay. In addition, the synthesis of MCP requires not only sufficient nitrogen source, but also carbon shelf provided by VFA and energy released by rumen fermentation. In the current research, the rumen PA concentration of PG and AG group was 58.4 and 55.5% higher than that of WG group, and the AA/PA ratio was 59.7 and 79.1% lower than that of WG group, respectively, indicating that peanut vine and alfalfa hay promoted rumen PA fermentation. It was reported that (38), ruminants fed a high fiber diet were beneficial to the rumen AA fermentation, and a diet in low fiber were beneficial to rumen PA fermentation, which is consistent with the current results. In VFA, AA provides oxidation energy for ruminants, while PA is the precursor of glucose synthesis and mainly used for the growth and reproduction of ruminants. Therefore, alfalfa hay and peanut vine were conducive to steers fattening. VFA produced by rumen fermentation can be used by rumen microbiota to synthesize FA for their own growth (39). We also analyzed the correlation between VFA and muscle FA deposition and found that rumen AA content was positively correlated with muscle SFA deposition and negatively correlated with MUFA deposition. These results indicated that rumen microbiota mainly use AA to synthesize C18:0 and C16:0 even carbon chain fatty acids, and PA to synthesize odd carbon atom fatty acids (39), which are absorbed by ruminants and could eventually affect the FA in the muscle. Consequently, ruminant diets with different roughages may cause changes in rumen fermentation with some potentially favorable effects on meat quality.

In the present study, the similar richness estimates and diversity indices indicated that the rumen microbial diversity was similar among the three diet groups. However, the PCoA examining the phylogenetic divergence among the OTUs showed cluster between AG and PG group, and the WG group distinct separation from AG and PG group, indicating that dietary interventions can impact the rumen microbial community (8). Most of the bacteria identified were phylum *Bacteroidetes* and *Firmicutes*, followed by phylum *Proteobacteria*. The phylum *Proteobacteria* has highly diverse metabolic functions, which is dominant in neonatal periods, and then decline to the lowest proportion while that of *Bacteroidetes* becomes the highest (40). This can explain why phylum *Proteobacteria* represented 2–4% of total rumen bacteria in the three groups. In addition, phylum *Bacteroidetes* was more predominant than *Firmicutes* in the three groups, which was consistent with the result of Niu et al., who reported that the rumen pH was ~6.74 and the phylum *Bacteroidetes* proportion (57.98%) was higher than that of *Firmicutes* (35.2%), followed by *Proteobacteria* (1.96%), and predicted the phylum *Bacteroidetes* maybe predominant instead of *Firmicutes* in the normal pH range (35). However, compared with AG and WG groups, PG group had a lower proportion of phylum *Bacteroides* (46%) and a higher proportion of phylum *Firmicutes* (43%), which may be due to the lower rumen pH value in PG group and result in a substantially decreased proportion of *Bacteroidetes* and an increased proportion of *Firmicutes* in the rumen microbial community (41). The phylum *Tenericutes* has strict requirements on nutrition, and dietary treatment may affect its abundance. Lignin in wheat straw was not easy to digest, which may be the reason for the decrease of phylum *Tenericutes* abundance in WG group. The abundance of phylum *Tenericutes* also decreased in AG group, which may be related to the bioactive substances in alfalfa, such as saponins. Alfalfa saponins can increase the reproduction rate of rumen protozoa (42). At the same time, there is a predatory relationship between rumen protozoa and rumen bacteria, which can change the structure of rumen microbial community (43). Within phylum *Bacteroidetes*, the dominant genera of three dietary treatments were composed of *Prevotella* and *Bacteroidetess*, among which *Prevotella* accounted for the largest proportion, consistent with a previous study (44). Previous studies reported that *Bacteroidetes* was associated with the degradation of proteins and carbohydrates (45). Thus, the abundances of *norank_f_Bacteroidales_BS11_gut_group* increased in AG group, because of the highest CP content in alfalfa hay. Interestingly, we also observed that the abundance of *Anaeroplasma* increased when steers consumed a diet containing peanut vine. To our knowledge, no studies are available that describe effects of the roughage diet on *Anaeroplasma* in ruminants. *Ruminococcaceae* is the most typical cellulolytic bacteria in the phylum *Firmicutes*, which can produce cellulase and hemicellulase to decompose plant fiber (46). Compared with WG group, the abundance of *Ruminococcaceae_UCG-001* decreased in AG group because of the lesser amount of substrate fiber available for them (35).The activity of cellulolytic bacteria was affected by rumen fluid pH, and the decrease of pH value would affect the decomposition rate of fiber (47). *Ruminococcus_2* and *Succinivibrionaceae_UCG-002* belonged to cellulolytic bacteria, which may be the reason why they are positively correlated with pH value. Under the action of intestinal bacteria, dietary fiber can produce short chain FA such as acetic acid, propionic acid, and butyric acid, which provide energy for fat metabolism, protein metabolism and carbohydrate metabolism (48). There was a positive correlation between the abundance of *Prevotella_ 1* and the concentrations of acetic acid, which was related to the fact that acetic acid was the main component in its decomposition products (49). Studies had shown that the content of acetic acid in the diet with high crude fiber content increases, and cellulose decomposing bacteria are conducive to the production of acetic acid (50). Actually, *Prevotella* could not degrade fiber directly, but can enhance the activity of cellulolytic bacteria when it is co-cultured with cellulolytic bacteria (51), and was closely related to the dietary fiber digestibility (52). In our study, wheat straw has higher crude fiber, the acetic acid content was the highest in WG group, while the abundance of *Prevotella_ 1* was also the highest, which indirectly supported *Prevotella_ 1* as an important synergist of fiber degradation. Propionic acid plays an important role in carbohydrate and protein metabolism, participates in the body function, and is conducive to maintaining the normal function of intestine (53). Studies in Sunit sheep that grazing alone and combined with barn feeding have shown that the concentrations of propionic acid were negatively correlated to the abundance of *Bacteroides*, and positively correlated to *Ruminococcus* (54). Contrary to the results obtained in this study, the concentrations of propionic acid were negatively linked to the abundance of *norank_f_Bacteroidales_RF16_group, Ruminococcaceae_UCG-001, Ruminococcaceae_UCG-011 and [Ruminococcus]_gauvreauii_group*. Notably, compared with feeding wheat straw, the abundance of *Ruminococcaceae_UCG-001* decreased significantly in steers fed with alfalfa hay, but the abundance of *Firmicutes* increased, and the content of propionic acid increased significantly, which indicated that alfalfa hay can selectively enhance the enrichment of some microbial communities in rumen. The main metabolites of *Lactobacillus* are lactic acid, while those of *Succinivibrionaceae* are acetic acid, succinic acid and a small amount of lactic acid (55). *Pseudobutyrivibrio* and *Butyrivibrio_ 2* are the main butyric acid producing bacteria (56). *Christensenellaceae* is beneficial to human health and can ferment polysaccharides to produce butyric acid (57). Therefore, this may explain why the abundance of *Lactobacillus* and *Succinivibrionaceae* are negatively correlated with the content of butyric acid, and *Pseudobutyrivibrio, Butyrivibrio_ 2*, and *Christensenellaceae* are positively correlated with the content of butyric acid in rumen. *Ruminococcus* generally ferments cellulose to produce acetic acid, but previous research found that *Ruminococcus* can produce butyric acid in pig intestine (58). In this study, *Ruminococcaceae_ NK4A214_ Group* belongs to *Ruminococcus* and has positive correlation with the content of butyric acid in the rumen. At present, there is no research to show whether the metabolism of *Ruminococcaceae_ NK4A214_ Group* in rumen is related to the production of butyric acid. In general, the relationship between physiological parameters and other rumen microorganisms needs to be further studied in the complex rumen microbial system.

The correlation analysis revealed that most nutrition intake, rumen fermentation, muscle FA deposition parameters were associated with the genera *norank_f_Bacteroidales_RF16_group* and *Ruminococcaceae_UCG-001*, which belong to the phyla *Bacteroidetes* and *Firmicutes*, respectively. In this study, the abundance of *Ruminococcaceae_UCG-001* was positively correlated with NDF and ADF intake, and negatively correlated with CP intake. Thus, we concluded that the *Ruminococcaceae_UCG-001* may play a role in the degradation of cellulose. The *norank_f_Bacteroidales_RF16_group* were found to be negatively correlated with CP intake and positively correlated with NDF and ADF intake in our study. Indeed, previous studies concluded that the relative abundance of *Bacteroidetes* in rumen of cattle in grazing environment was negatively correlated with NDF content in diet (44), which was inconsistent with the results of this study. The reason may be related to the diversity of forage species and rumen microbial. It is also the possible reason for the negative correlation between ruminal NH_3_-N concentration and the abundance of *norank_f_Bacteroidales_RF16_group*, ruminal MCP concentration and the abundance of *Ruminococcaceae UCG-001*. In addition, some studies have also shown that fat deposition is closely related to the proportion or relative abundance of *Bacteroides* and *Firmicum* (48). In this study, muscle SFA deposition were positively correlated to the abundance of *Ruminococcaceae_UCG-001*, while PUFA, *n*−6 PUFA, and *n*−3 PUFA deposition were negatively correlated to the abundance of *Ruminococcaceae_UCG-001*. Some scholars have found that bio-hydrogenated products are related to the existence of *Clostridiales* and *Ruminococcaceae* etc. (59), but the relationship between them has not been fully understood. In a recent study, a significant negative correlation between the content of C18:3 in mutton and the abundance of *Bacteroides* had been found (54). Of particular interest in our study that muscle n-3 PUFA deposition was negatively correlated to the abundance of *norank_f_Bacteroidales_RF16_group*, which suggested that *norank_f_Bacteroidales_RF16_group* may be the main microorganism responsible for the hydrogenation of linolenic acid in rumen. Relative to steers fed on wheat straw diets, using alfalfa hay as roughage source for steers decrease the abundance of *norank_f_Bacteroidales_RF16_group* in rumen, and have positive effects on the fatty acid profile of their meat (higher n-3 PUFA and PUFA, lower SFA), resulting in a healthier product. Dietary PUFA are extensively biohydrogenated in the rumen, in which unsaturated FA are isomerised and hydrogenated by ruminal microbes to form SFA. Alfalfa can produce protein-xanthophyll-rich (PX) that has 50% of its FA content as 18:3*n*−3 (60), which may have some protection from rumen biohydrogenation, and increase the *n*−3 PUFA content of beef (61). As an aside, we note that more data have recently been used to study rumen microbiota using molecular methods, but further research on other rumen microbes, such as protozoa, archaea and fungi, is needed to improve our understanding of the interaction between FA and rumen microbiota.

## Conclusions

In conclusion, compared with wheat straw and peanut vine, alfalfa hay could improve the average daily gain and reduce feed/gain ratio, and promote muscle marbling score, *n*−3 PUFA deposition, rumen NH_3_-N and MCP concentration of Simmental crossbred steers. Therefore, alfalfa hay provides better fattening effect on steers. Most nutrition intake, rumen fermentation, muscle FA deposition parameters were associated with the genera *norank_f_Bacteroidales_RF16_group* and *Ruminococcaceae_UCG-001*. Alfalfa rich in n-3 PUFA would selectively alter some rumen bacterial colonization and reduce the abundance of *Ruminococcaceae_UCG-001* involved in hydrogenation, increase the rumen protective effect of C18:3*n*−3, which is beneficial to the deposition of muscle *n*−3 PUFA and PUFA.

## Data Availability Statement

The datasets presented in this study can be found in online repositories. The names of the repository/repositories and accession number(s) can be found in the article/Supplementary Material.

## Ethics Statement

The animal study was reviewed and approved by Animal Welfare and Ethics Committee of Henan Agricultural University (approval number: HENAU-2018-039).

## Author Contributions

XZ, BL, and JX performed experiments and analyzed data. SZ and MH participated in the data collection. MG assisted with animal experimentation. YC, DL, CW, and SM provided advice in design and performance of experiments. XZ wrote the manuscript draft. YS supervised the study. All authors read and approved the final manuscript.

## Funding

Financial support for this research was provided by China Agriculture Research System of MOF and MARA (CARS-34), Science and Technology Innovation Team of Henan Province High Quality Forage and Animal Health (22IRTSTHN022), and Major Public Welfare Projects in Henan Province (201300110400).

## Conflict of Interest

The authors declare that the research was conducted in the absence of any commercial or financial relationships that could be construed as a potential conflict of interest.

## Publisher's Note

All claims expressed in this article are solely those of the authors and do not necessarily represent those of their affiliated organizations, or those of the publisher, the editors and the reviewers. Any product that may be evaluated in this article, or claim that may be made by its manufacturer, is not guaranteed or endorsed by the publisher.
