# Supplementary figures and images for: Effects of Different Roughage Diets on Fattening Performance, Meat Quality, Fatty Acid Composition, and Rumen Microbe in Steers
Source: Front Nutr. 2022 Jun 21;9:885069. doi: 10.3389/fnut.2022.885069 (PMC9253607; doi:10.3389/fnut.2022.885069)

Supplementary Material

# Supplementary Figures

**
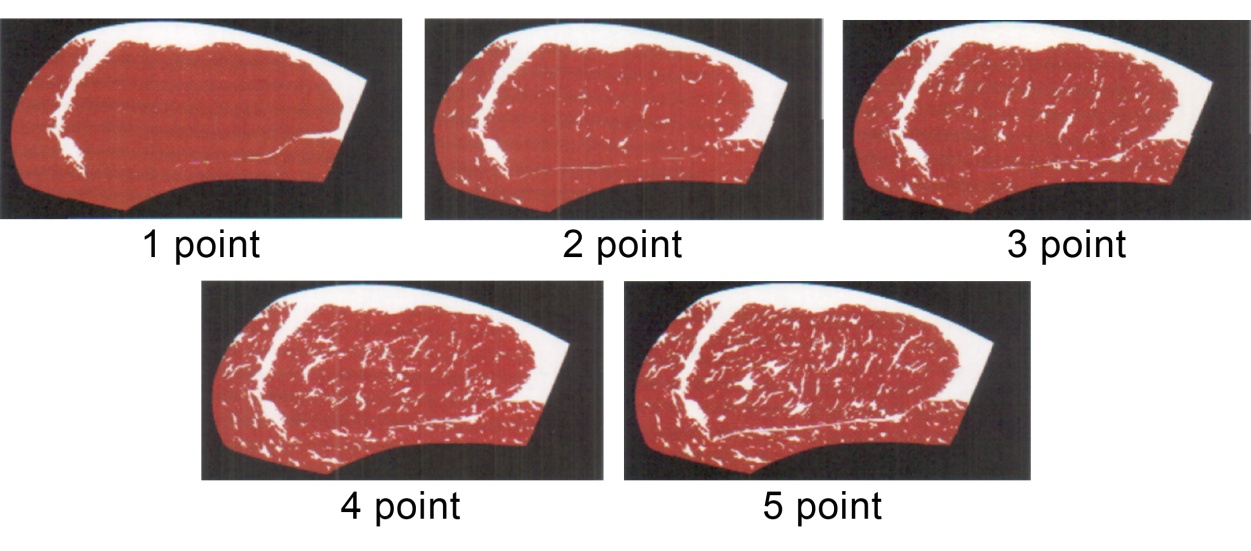
**

**Supplementary Figure 1.** Marble score standard

Supplement: Supplementary file 1 [file Table_1.DOCX]
